# Review on Emotion Recognition Based on Electroencephalography

**DOI:** 10.3389/fncom.2021.758212

**Published:** 2021-10-01

**Authors:** Haoran Liu, Ying Zhang, Yujun Li, Xiangyi Kong

**Affiliations:** ^1^The Boiler and Pressure Vessel Safety Inspection Institute of Henan Province, Zhengzhou, China; ^2^Patent Examination Cooperation (Henan) Center of the Patent Office, CNIPA, Zhengzhou, China

**Keywords:** emotion recognition, EEG, convolution neural network, DEAP, SEED, DREAMER

## Abstract

Emotions are closely related to human behavior, family, and society. Changes in emotions can cause differences in electroencephalography (EEG) signals, which show different emotional states and are not easy to disguise. EEG-based emotion recognition has been widely used in human-computer interaction, medical diagnosis, military, and other fields. In this paper, we describe the common steps of an emotion recognition algorithm based on EEG from data acquisition, preprocessing, feature extraction, feature selection to classifier. Then, we review the existing EEG-based emotional recognition methods, as well as assess their classification effect. This paper will help researchers quickly understand the basic theory of emotion recognition and provide references for the future development of EEG. Moreover, emotion is an important representation of safety psychology.

## Introduction

Emotions are not only physiological states of the various feelings, thoughts, and behaviors of integrated humans but also psychological and physiological reactions produced by various external stimulation. Emotions occupy an important position in daily life and work. It is significant to recognize emotions correctly in many fields. Recently, the study of emotion recognition is mainly used in psychology, emotional calculation, artificial intelligence, computer vision, and medical treatment, etc. (Ramirez et al., 2010; Xin et al., 2019; Fürbass et al., 2020). For example, emotion recognition is helpful to the diagnosis of depression, schizophrenia, and other mental diseases. It can assist doctors to understand the true emotions of patients. Furthermore, emotion recognition by computers can bring human satisfactory user human-computer interaction experience.

In recent years, existing emotion recognition models were classified into two categories, the methods based on physiological signals and the methods based on non-physiological signals. Compared to non-physiological signals, physiological signals are not susceptible to subjective factors, which can show human emotional states truly. Therefore, emotion recognition based on physiological signals has great advantages in reliability and practicality. Current concerns of scholars concentrate on physiological signaling at present. In emotion recognition, the physiological signals include EEG, facial expression, Eye Movement (EM), Electrocardiogram (ECG), and so on. We can judge the true emotions of the participants correctly according to these physiological signals. In the field of research based on physiological signals, EEG is a spontaneous, non-subjective physiological signal, which can objectively reflect human emotional states (Mohamed et al., 2018). Therefore, EEG-based emotion recognition has become an important research topic. Niemic (2004) verified EEG played a key role in emotion study and illustrated the activity of different brain regions was closely related to some kind of emotional states.

EEG-based emotion recognition methods are mainly developed from two aspects: traditional machine learning and deep learning. In emotion recognition methods based on traditional machine learning, features are extracted manually to input to Naive Bayes (NB), Support Vector Machine (SVM) and other classifiers to classify and recognize. The emotion recognition methods based on deep learning automatically learns deep features and recognizes emotions through such models as Long Short-Term Memory (LSTM) and Recurrent Neural Network (RNN), thus effectively simplifying the process of feature extraction. Lin et al. (2018) introduced the overall process of the traditional machine learning method for EEG emotion recognition, including emotion trigger, signal acquisition, feature extraction, and classification recognition, etc. At the same time, the problem of traditional machine learning methods was revealed, which clarified the future direction in EEG emotion recognition. Since the EEG signals have the characteristics of being non-linear and high-dimensional, it is not easy to distinguish EEG signals with a linear algorithm. Deep learning can realize end-to-end mapping, which is helpful to solve non-linear problems. Craik et al. (2019) given the effect of emotion recognition by Deep Belief Network (DBN), RNN, and Convolutional Neural Network (CNN). The superiority of deep neural networks in the EEG classification tasks had been verified.

In recent years, CNN (Yea-Hoon et al., 2018; Dm et al., 2021; Keelawat et al., 2021), LSTM (Li et al., 2017; Liu et al., 2017; Sharma et al., 2020), Generative Adversarial Network (GAN) (Luo, 2018), and other network models have been widely used in EEG emotion recognition. In this paper, several selected keywords were used to search related literature in Elsevier and Springer and 645 published studies were retrieved. Among them, 102 were selected for review after removing duplicates and incongruent literature. The selected keywords are: “EEG emotion recognition” or “deep learning” or “classification,” “EEG emotion classification” or “machine learning,” and “emotion recognition” or “EEG feature extraction.”

## Overview

Comprehension of the concepts and features of EEG signals, emotion induction methods, and common emotion classification models are necessary for emotion recognition based on EEG. The following section provides a detailed introduction to each of these aspects.

### Electroencephalography Signal

**FIGURE 1:**
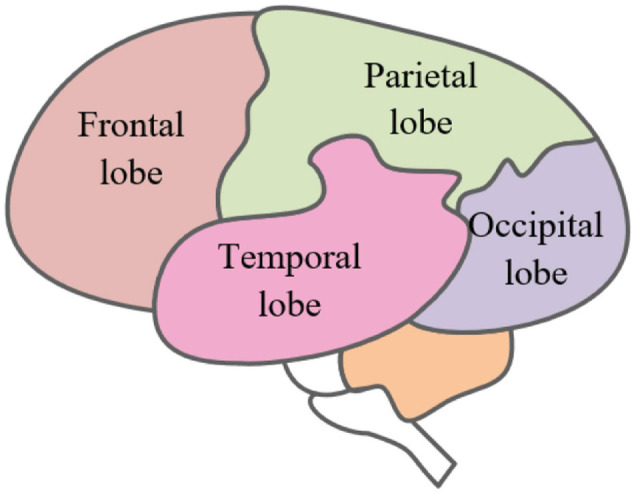
Differential brain map.

As the most important organ and tissue in the human body, the brain plays a key role in the stability of the central nervous system (Chen and Mehmood, 2019), which can control and regulate the body’s advanced functions such as learning, communication, and thinking. In EEG emotion recognition, it is particularly important for correctly recognizing the functions of the major brain parts. The brain is mainly divided into three parts, which are the brain nucleus, brain margin, and cerebral cortex. The cerebral skin is also the most functional and advanced part of the brain. It can be divided into four parts in space, which are the parietal lobe, frontal lobe, temporal lobe, and occipital lobe, as shown in Figure 1. Different regions control different functions. The regions mutually cooperate to control people’s daily behavior activities (Kyanamire and Yang, 2020).

The frontal lobe is located in the former region of the central sulcus of the brain. It includes all the advanced features and controls human emotional expression and thinking. The parietal lobe is located in the middle of the brain, which is the primary sensory area of the body. It is responsible for perceiving touch pressure, temperature, sense of taste, and pain. The temporal lobe is located below the brain sylvian fissure, which is an auditory area. It is treated to external auditory information and has a certain association with the memory and emotion of the body. Located at the back of the skull, the occipital lobe is the visual processing center of the body and can process visual information such as color, light and shade, and motion speed. It is essential in the integration process of received information.

Among various physiological signals, EEG signals are spontaneous and difficult to camouflage and can reflect the interaction between the brain and other parts. They can also show the specific state of the brain. EEG plays a critical role in emotional identification research (Eo et al., 2020).

EEG often shows rhythmic features. According to its frequency range, EEG is generally divided into five basic bands (Sarno et al., 2016; Thammasan et al., 2017; Zhuang et al., 2017; Liu Y. et al., 2020). The frequency of δ rhythm is usually between 0.5 and 3 Hz and the amplitude of that is about 20–200 μ*V*. The EEG of the mentioned frequency band appears when people are in a state of drowsiness and very tired. The frequency of θ rhythm is between 4 and 8 Hz and the amplitude is approximately 10–50 μ*V*, which occurs under stress. The frequency of α rhythm is between 9 and 13 Hz and the amplitude is about 20–100 μ*V*. The frequency of β rhythm is between 14 and 30 Hz and the amplitude is about 5–20 μ*V*. It appears when the human brain is excited. The frequency of γ rhythm ranges over 31 Hz and appears when human attention is highly concentrated or for some perceived behavior.

### Emotion Recognition Model

Psychologists believe that emotion is a subjective attitude generated by a person’s experience of external things, as well as an instinctive coordinated response made by the body, which may include the joint effects of language, behavior, and spirit (Behm et al., 2002). Thoits (Cabanac, 2002) interpreted emotions as a continuous process of subjective feelings. At present, the definition of emotions has not been unified. In many cases, emotions are usually associated with the personality, mood, and desire of an individual person (Kumar and Kumar, 2015; laza-Del-Arco et al., 2019). The division and induction of emotion recognition models will be specifically described as follows.

#### Emotion Model

**FIGURE 2:**
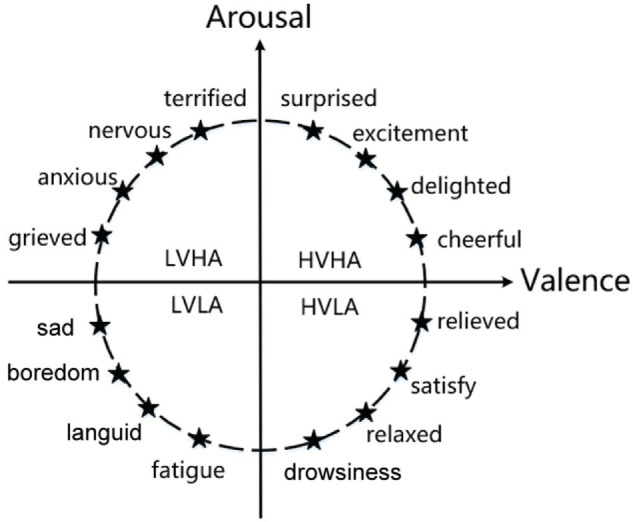
The emotion model of valence and arousal.

The emotion recognition model plays a key role in emotion recognition research, which includes discrete models and continuous models. The discrete model theory outlines that people’s emotions are composed of basic emotions. In one study (Plutchik, 2001), emotions were categorized into eight basic states, love, anger, sadness, joy, expectation, hatred, fear, and surprise. All human emotions can be formed through a combination of one or more of these basic emotions. Ekman et al. (1987) divided basic emotions into fear, anger, sadness, and likes. The continuous model theory is a dimensional theory proposed by Lang et al. (1990) and Russel (2003). It classifies emotions from dimensional space. The dimensional theory outlines that emotion is constantly changing, such as the two-dimension emotion model composed of valence and arousal and the three-dimension emotion model composed of valence, arousal, and dominance. As shown in Figure 2, in the two-dimension emotion model, the ordinate represents arousal, and the abscissa represents valence. Different emotions can be represented by different coordinate positions in the figure. Continuous model theory can divide more emotion statuses and distinguish between different emotions more intuitively.

#### Emotion Induction

In emotion recognition, we need to acquire the EEG signals of the corresponding emotional state, which inspires the corresponding emotional state to acquire the recorded EEG signals. There are currently several mainstream emotion methods, such as picture induction, video induction, and music induction.

Picture induction (Constantinescu et al., 2017) uses different pictures to inspire different emotion states and then records participants’ EEG signals. The pictures are required to have some significant emotional features or produce strong stimulation, such as the well-known International Affective Picture System (IAPS), which has a significantly different emotional expression after strict screening and which provides helpful assistance in emotional research.

Video induction (Soleymani et al., 2014; Huo et al., 2016; Zheng and Lu, 2016; Hu et al., 2020) uses different types of videos for emotion induction. Compared with picture induction, video induction requires a shorter time to obtain different emotions and has more obvious effects. However, different facial expressions appear when the subjects watch the videos in video induction, which will produce a large number of different noise interference, such as Electromyography (EMG) and Electrooculogram (EOG) which increases the difficulty of experimental data preprocessing.

Music induction (Soleymani et al., 2013; Greco et al., 2017) needs a quiet environment and the subjects are required to listen to different types of music. The music is then associated with corresponding emotional states reached whilst listening, such as joy, calmness, sadness, and so on. However, music induction is not suitable for everyone. It requires subjects to accurately experience the rhythm of the music. For people who have little contact with music, music induction cannot achieve consistent results.

## Method

In the task of emotion recognition based on EEG signals, the collected EEG signals need to be pre-processed, which involves feature extraction, feature selection, and classification, as described in the section that follows.

### Datasets

Many researchers have recently carried out relevant experiments and published several open datasets of affective computing. In emotion recognition, the public datasets based on EEG are DEAP (Database for Emotion Analysis using Physiological Signals), SEED, and DREAMER.

DEAP dataset (Verma and Tiwary, 2014) is a multi-channel dataset that is used to study human emotional states. The dataset includes a variety of physiological signals such as physiological signals, psychological scales, and facial expressions. These physiological signals are recorded by 32 subjects by watching a 40-min music video. The dataset includes 32-channel EEG, 2-channel EOG, and 2-channel EMG. A 3-s silence time was set before each signal is recorded. This dataset can be used to study physiological signals under multi-modality.

**FIGURE 3:**
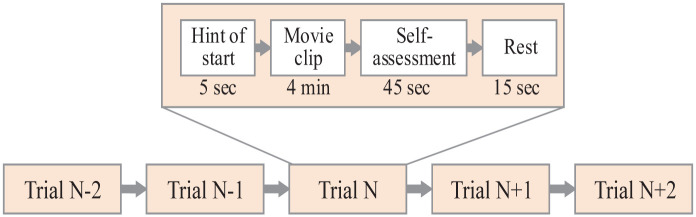
The collection process of the SEED dataset.

SEED dataset (Duan et al., 2013; Zheng and Lu, 2015) is an EEG dataset collected by the Brain-like Computing and Machine Intelligence laboratory (BCMI). The experiment recorded EEG from 15 subjects. Each participant was asked to watch 15 clips of the film three times. The detailed process of the SEED collection is shown in Figure 3. The dataset has 62 channels and its sampling frequency is 200 Hz.

The DREAMER (Katsigiannis and Ramzan, 2017) dataset, published by the University of the West of Scotland, used 18 movie clips as stimuli to evoke emotion. The experiment recorded 14 channels of EEG from 23 subjects, who also self-evaluated their emotion including valence, arousal, and dominance. The sampling frequency was 128 Hz.

### Preprocessing

Original EEG signals are a series of curves that change over time. Due to the interference generated by the EEG device itself or the transmission line itself, original data includes many noises and interference in the process of acquisition, which affect the classification. To improve the classification performance, the original EEG signal should be denoised and deinterfered. After a reasonable preprocessing, a relatively clean signal is obtained (Pedroni et al., 2019). EEGLAB is a popular preprocessing toolkit for EEG data (Delorme and Makeig, 2004). The preprocessing of EEG original signal included channel location, filtering, baseline correction, independent principal component analysis, and so on.

After the original EEG data importing into EEGLAB, we can use filtering to suppress the noise of the signal. Then, we use Butterworth band-pass filter to remove the electromagnetic interference. There are different kinds of artifact interference in EEG, such as eyeball movements and eye movements from blinking, muscle artifacts from muscle extension or contraction, ECG artifacts from heartbeat expansion and contraction, and power frequency interference. Although filtering can remove most of the noise, it is still difficult to remove all the artifacts mentioned. This section lists some ways that artifacts can be removed (Maiorana et al., 2016).

#### Regression Method

The recorded EEG is composed of true EEG signals and artifacts. A regression filter is used to calculate the proportion of reference signals in a single EEG channel based on the reference channel constructed by EOG. Then, these artifacts are removed from contaminated EEG by regression method (Wallstrom et al., 2004). The regression method is the most commonly used method for removing EOG artifacts.

#### Adaptive Filtering Method

Adaptive filtering is used to eliminate the EOG. He et al. (2004) used recursive least-mean-square adaptive filtering to remove EOG. Adaptive filtering can effectively remove multiple EOG artifacts with stability and fast convergence.

### Feature Extraction

After EEG signal preprocessing, it is necessary to extract and select the features of the preprocessed signals. Feature extraction refers to the process of transforming the signal, separating the relevant signal features from the irrelevant components, calculating the features related to the target task, and expressing them in a compact or meaningful form. Feature selection can improve the performance of emotion recognition by selecting the most representative feature subset, meaning the process of extraction and the selection of EEG features is more important than anything else. We introduced the EEG-based emotion feature extraction as follows.

#### Time Domain Features

Time domain feature extraction is used to extract the statistical parameters of EEG from the time domain as the features of EEG. Time domain analysis mainly describes the waveform features of EEG signals. From the perspective of the time domain, we can extract time domain features such as Zero Crossing Rate (ZCR), Slope Sign Change (SSC), and Willison Amplitude (WAMP) from EEG signals (Riedl et al., 2013; Namazi et al., 2019). These representative time domain features are described in the following chapters.

1. Zero Crossing Rate

Zero Crossing Rate is the number that the frequency of the EEG waveform passes through the zero axis in unit time, and can be used to map the spectral information. To reduce the effect of random noise on ZCR, the threshold ε is introduced. Let *x*_*i*_(*t*) and *x*_*i* + 1_(*t*) denote the EEG signal samples obtained by continuous sampling, if Eqs 1, 2 are satisfied, the ZCR will increase.


(1) $$\left\{x_{i}\left(t\right)<0\;and\;x_{i+1}\left(t\right)>0\right\}or\left\{x_{i}\left(t\right)>0\;and\;x_{i+1}\left(t\right)<0\right\}$$

(2) $$\left|x_{i}\left(t\right)-x_{i+1}\left(t\right)\right|\geq \varepsilon$$

1. Slope Sign Change

Slope Sign Change is the number of times that the slope of the recognition waveform changes its sign in definition, which reflects the frequency features of the EEG signal indirectly. To reduce the influence of random noise on SSC, the appropriate threshold ε should be set. For a given continuous sample *x*_*i*_(*t*), *x*_*i* + 1_(*t*), and *x*_*i*−1_(*t*), if Eqs 3, 4 are satisfied, the SSC will increase.


(3) $$\{x_{i}\left(t\right)<x_{i+1}\left(t\right)andx_{i}\left(t\right)<x_{i-1}\left(t\right)\}or\{x_{i}\left(t\right)>x_{i+1}\left(t\right)\;andx_{i}\left(t\right)>x_{i-1}\left(t\right)\}$$

(4) $$\left|x_{i}\left(t\right)-x_{i+1}\left(t\right)\right|\geq \varepsilon \;and\;\left|x_{i}\left(t\right)-x_{i-1}\left(t\right)\right|\geq \varepsilon$$

1. Willison Amplitude

Willison Amplitude means the number of times the difference value between the absolute values of amplitudes of two consecutive EEG samples exceeds the predetermined threshold. It reflects the variation law of the amplitude of EEG signals. It can be calculated according to Eqs 5, 6.


(5) $$WAMP=\sum_{i=1}^{N-1}f\left(x\left(t\right)\right)$$

(6) $$f\left(x\left(t\right)\right)=\left\{ \begin{array}{c} 1,\left|x_{\text{i}}\left(t\right)-x_{i+1}\left(t\right)\right|>\varepsilon \\ 0,\mathrm{otherwise} \end{array}\right.$$

where *N* is the length of the signal, *x*_*i*_(*t*) and *x*_*i* + 1_(*t*) represents the samples obtained by continuous sampling, and ε is the threshold.

#### Frequency Domain Features

In comparison with time domain analysis, frequency domain analysis can reveal the various components of the signal frequency. The frequency domain features mainly include power spectrum, approximate entropy (ApEn), and sort entropy. The basic process of frequency domain analysis is described as follows. Firstly, the EEG signal is separated into five rhythm signals. Then, we extract each feature of the rhythmic signal by using Fourier transform. The exemplified elaboration of the typical brain frequency domain features is described as follows.

1. Power spectrum

Power spectrum estimation (Zhou et al., 2013) is a tool for estimating the power spectral density (PSD) of signals. It turns the original signal into a power spectrum that changes with frequency. The frequency components of the signal can be observed clearly and intuitively. The most common method of power spectrum estimation is classic spectrum estimation (Xin and Qv, 2010), which is achieved by Fourier transform. Let EEG signal be *x*(*t*), its autocorrelation function is *r*(*k*), the power spectral density function *P*(ω) is defined as:


(7) $$P\left(\omega \right)=\sum_{k=-\infty }^{+\infty }r\left(k\right)e^{-j\omega k}$$

The most common method of classic spectrum estimation is the direct method (periodical graphic), which gives a periodic spectrum estimate by Eq. 8.


(8) $$\overset{\wedge }{P}\left(\omega \right)=\frac{1}{N}{\left|\sum_{t=1}^{N}x\left(t\right)e^{-j\omega t}\right|}^{2}$$

where *N* is the signal length. Although the direct method can improve the resolution of the power spectrum, the variance is large, and there may be a random fluctuation for too long a length of signal.

1. Approximate entropy

ApEn is a typical method of quantifying the complexity of finite length physiological signals (Namazi et al., 2019). The larger ApEn is, the stronger and higher the randomness and complexity of the time series are. The calculation process of approximate entropy is described as follows.

For the original signal *x*(*t*), the signal length is *N*(1≤*t*≤*N*). A signal *x*(*t*) is turned into high-dimension feature space to obtain a sequence of *m*-dimension vectors, that is


(9) $$X_{m}\left(t\right)=\left\{x\left(t\right),x\left(t+1\right),\;\ldots \;,x\left(t+m-1\right)\right\}$$

Let $d_{tk}^{m}$ indicate the maximum distance between two vectors, where *k* = 0,1,…,*m*, that is


(10) $$d_{tk}^{m}=d\left[X_{t}^{m},X_{k}^{m}\right]=\max\left(\left|x\left(t+z\right)-x\left(j-z\right)\right|\right)$$

Let ε represent a fixed threshold and $C_{t}^{m}\left(\varepsilon \right)$ indicates the probability of distance between the vector *X*_*m*_(*t*) and *X*_*m*_(*k*), that is


(11) $$C_{t}^{m}\left(\varepsilon \right)=\frac{\sum_{k=1}^{N-m+1}\theta \left(d_{tk}^{m}-\varepsilon \right)}{N-m+1}$$

where *t* = 1,2,…,*N*−*m* + 1, $C_{t}^{m}\left(\varepsilon \right)$ less thanε. θ(*x*) can be defined as


(12) $$\theta \left(x\right)=\left\{ \begin{array}{c} 1,x\geq 0\\ 0,x<0 \end{array}\right.$$

Let ϕ^*m*^(*ε*) represents the average of the logarithms of $C_{t}^{m}\left(\varepsilon \right)$, as shown in Eq. 13.


(13) $$\phi^{m}\left(\varepsilon \right)=\frac{\sum_{t=1}^{N-m+1}\ln C_{t}^{m}\left(\varepsilon \right)}{N-m+1}$$

when the dimension increases to *m+1*, the above operation is repeated to obtain ϕ^*m* + 1^(*ε*), that is


(14) $$\phi^{m+1}\left(\varepsilon \right)=\frac{\sum_{t=1}^{N-m}\ln C_{t}^{m+1}\left(\varepsilon \right)}{N-m}$$

The approximate entropy at this moment is the difference between ϕ^*m*^(*ε*) and ϕ^*m* + 1^(*ε*), that is


(15) $$ApEn\left(m,\varepsilon ,N\right)=\phi^{m}\left(\varepsilon \right)-\phi^{m+1}\left(\varepsilon \right)$$

The approximate entropy is often affected by parameter ε and parameter *N*. The value of the parameter ε is approximately 0.1 SD ∼0.2 SD, where SD represents the standard deviation.

1. Permutation entropy

Similar to approximate entropy, permutation entropy (PeEn) is also a measurement algorithm using the complexity of time series with advantages of strong anti-interference ability and fast computation speed (Riedl et al., 2013). It has been widely used in speech detection, epileptic EEG classification, and other fields.

The original signal can be denoted as *x*(*t*) (1≤*t*≤*N*), and the signal *x*(*t*) is embedded into the high-dimension feature space to construct the *m*-dimension vector *X*_*t*_, that is


(16) $$X_{t}=\left\{x\left(t\right),x\left(t+\tau \right),\;\ldots \;,x\left(t+\left(m-1\right)\tau \right)\right\}$$

where *t* = 1,2,…,*N*−*m* + 1, and τ is the delay time.

In *m*-dimension space, vector *X*_*t*_ is sorted in ascending order, and we can get *m*! kinds of sorting methods. Let the sorting mode of *X*_*t*_ be π_*k*_, and*k* = 1,2, …, *m*!. The appearing probability of π_*k*_ is *p*(π_*k*_), then


(17) $$p\left(\pi_{k}\right)=\frac{\sum_{k=1}^{N-m+1}\left\{\pi_{k}\right\}}{m!\left(N-m+1\right)}$$

The permutation entropy of the time series can be expressed as


(18) $$PeEn\left(m\right)=-\sum_{k=1}^{m!}p\left(\pi_{k}\right)\log p\left(\pi_{k}\right)$$

Considering the uncertainty and disorder of EEG signals, the value of parameter m ranges from 3 to 10. The permutation entropy intuitively reflects the complexity of the signal. The smaller the permutation entropy is, the more regular the signal is. On the contrary, the signal disorder is higher.

#### Time-Frequency Domain Features

Since the collected EEG signals are unstable, with the development of EEG analysis, only analyzing the signal in the time domain or frequency domain cannot extract the feature information at present. Features of the time-frequency domain extracted for EEG analysis can be used for comprehensive analysis (Toole, 2013; Alazrai et al., 2018).

In various signal processing, time-frequency analysis uses a variety of time-frequency transformation tools to interpret a time series simultaneously in the frequency domain and the time domain. This not only provides a way to expand the angle of signal analysis but also deepens people’s knowledge and understanding of the signal description. The common time-frequency analysis tools include Hilbert transform and Short-time Fourier transform (STFT) (Koenig and Dunn, 2005), etc. The basic process of time-frequency analysis is described as follows. Firstly, the time-frequency analysis tool is used and the signal of amplitude varying from time is converted to a time varying from frequency. Then, the time-frequency domain features are extracted by feature extraction tools. The typical time-frequency features include wavelet entropy and wavelet package coefficient entropy (Gao Q. et al., 2020). The wavelet entropy (Rosso et al., 2001) specifically is described as follows.

Original EEG is decomposed into *n*-layer by wavelet transform and several different frequency components are obtained. Let *N* denote the signal length, *x*(*t*) denotes the original signal, *E*_*k*_ denotes the energy of each frequency component of each node *k*, that is


(19) $$E_{k}=\sum_{j=1}^{L_{k}}d{\left(j\right)}^{2}$$

where *t* = 1,2,…,*N*. *j* is the summation exponent of the signal at each node *k*, *L*_*k*_ is the number of coefficients.

The total frequency energy of the signal *x*(*t*) is *E*_*total*_, which can be expressed as


(20) $$E_{total}=\sum_{k}E_{k}=\sum_{k}\sum_{j=1}^{L_{k}}d_{k}{\left(j\right)}^{2}$$

The relative wavelet energy *P*_*k*_ is available from the above steps, that is


(21) $$P_{k}=\frac{E_{k}}{E_{total}}=\frac{E_{k}}{\sum_{k}E_{k}}=\frac{\sum_{j=1}^{L_{k}}d{\left(j\right)}^{2}}{\sum_{k}\sum_{j=1}^{L_{k}}d_{k}{\left(j\right)}^{2}}$$

According to the principle that the sum of the wavelet energy is 1, the wavelet entropy can be expressed as


(22) $$S_{WaEn}=-\sum_{k}p_{k}\ln\left(p_{k}\right)$$

Wavelet entropy is a similar concept of information entropy constructed according to the wavelet transform, which can describe the energy features more accurately in the time-frequency domain. It also reveals the sparsity of the degree of the wavelet transform coefficient matrix.

#### Deep Domain Features

Deep learning does not need to manually extract features, and can automatically filtrate data and extract the high-dimension features of data. In addition, the deep neural network can be used to process EEG signals to train feature extraction models and perform classification or regression tasks at the same time (Roy et al., 2019). Pre-training of deep neural networks is used to fine-tune specific EEG tasks aiming for providing better initialization or regularization effects. Controlling its complexity is the primary goal of regularization so that it can achieve better generalization performance and enhance the robustness of the network. Weight decay, early stopping, dropout, and label smoothing are the most common regularization methods.

**FIGURE 4:**
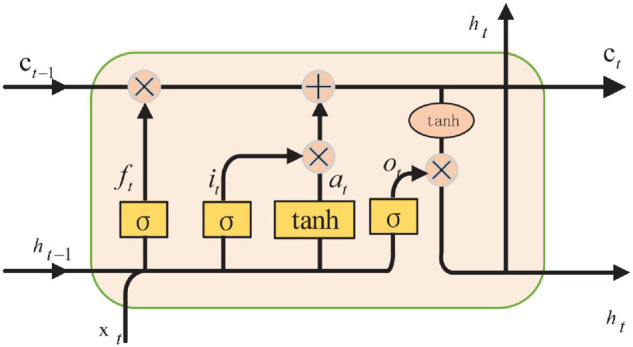
The structure of LSTM.

The recurrent neural network is applied frequently as network architecture. After improving on RNN (As et al., 2021), a network called the Long Short-Term Memory network is created. LSTM can learn which information is important and which information should be deleted from memory. Forget gate, input gate, and output gate are the parts of LSTM, as shown in Figure 4.

In LSTM, the first step is to determine which information can be left according to the output of the previous moment and the input of this time, which can be controlled by the sigmoid from the forget gate, as shown in Eq. 23.


(23) $$f_{t}=\sigma \left(W_{f}\cdot \left[h_{t-1},x_{t}\right]+b_{f}\right)$$

The next step is to generate new information which needed to be updated. From the input gate layer, the needed update values are determined by sigmoid. They then generate new candidate values to add using the tanh layer, so that unnecessary information can be removed. Eq. 24 is the process of adding new information.


(24) Ct=ft*Ct-1+it*Ct∼t

Finally, the output of the model can be calculated. The initial output is obtained through the sigmoid layer first, that is


(25) $$o_{t}=\sigma \left(W_{o}\left[h_{t-1},x_{t}\right]+b_{o}\right)$$

Then, the value is scaled to the value between –1 and 1. The product of and the value is the output of the model, that is


(26) $$h_{t}=o_{t}^{*}\tanh\left(C_{t}\right)$$

### Feature Selection

Feature selection can simplify the model and make a model more lucid. It also can speed up the training time, avoid dimension disaster and enhance the generalization ability by reducing overfitting (Jenke et al., 2017).

Principal component analysis (PCA) (Shlens, 2003) occupies the widest area of application among many data dimensionality reduction algorithms. The core of PCA is to map *n*-dimension features to *k*-dimension features. The *k*-dimension well as main component features are new orthogonal features as well as main components. Depending on the original *n*-dimension features, they are reconstructed. Assuming there are *n* rows of *m*-dimension data, the solving steps of PCA are shown as follows: (1) make the original data form *n*-row and *m*-column matrix named *X*; (2) each row of *X* subtracts the mean of this row; (3) the result of the covariance matrix $C=\frac{1}{m}XX^{T}$ is achieved; (4) calculate the covariance matrix’s eigenvalues and its corresponding eigenvectors; (5) arrange the eigenvectors from top to bottom into a matrix according to the value of the corresponding eigenvalues. Take the top *k* rows to form the matrix; (6) the data after dimension reduction is turned to *k*-dimension by *Y=PX*.

Independent component analysis (ICA) (Amari, 2001) is a way of finding potential factors or components from multi-dimension data. It converts random multivariate signals into independent components, which works to remove artifacts from the EEG signal. It indicates that information executed by one component is unable to be indirectly related to other components. Therefore, ICA can extract features from mixed signals.

Linear discriminant analysis (LDA) (Schlögl et al., 2009) is a supervised data dimensionality reduction method that is used to identify a given data pattern. It can be used either for dimensionality reduction or classification. The basic idea of LDA is to project the sample data onto a straight line and make the projection points after the projection as close as possible, and make the intra-class gap as small as possible and the inter-class gap as large as possible. Then the new sample category is determined. It is up to the position of projection points.

### Classifier

After selecting the feature that provides the best classification accuracy, the selected features are sent to the classifier to achieve classification. A classifier can draw a boundary between two or more categories and then label the category based on the features it chooses. The boundary can be regarded as a separate hyperplane belonging to a multidimensional feature space. In a word, the better the classifier, the better the hyperplane, and the larger the distance from all categories. These features are further classified by using machine learning or a deep network to group similar features into one category. Common machine learning classifiers include support vector machine (SVM), K-means clustering (K-means), K-nearest neighbor (K-NN), and Random forest (RF) (Alarcão and Fonseca, 2017).

**FIGURE 5:**
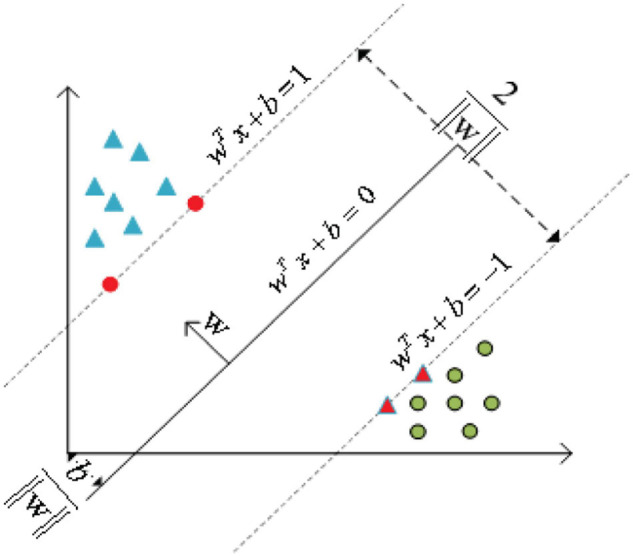
Principle of support vector machine.

As shown in Figure 5, the basic principle of SVM (Smola and Schölkopf, 2004; Zhang et al., 2016) can be summarized as mapping the indivisible data to the high-dimension space, then finding the hyperplane that classifies the data correctly and takes the distance from this plane to all the data to the maximum.

K-means clustering (Asghar et al., 2019; Wagh and Vasanth, 2019) is a kind of unsupervised learning method, which is mainly used to solve clustering problems. It is a simple iterative clustering algorithm, which separating the nearest mean of the sample points constantly. This algorithm divides a given sample set into clusters based on clustering centers, where the center of each class is obtained according to the mean of all the values in the class. This process is achieved by minimizing the Euclidean distance between the sample point and the cluster center. The association of each classification result belongs to a given cluster with the nearest cluster center and then repeats it in each iteration to achieve a new cluster and calculate the new cluster center. For given data *X* and categories number *k*, the minimization formula that minimizes the sum of clustering squares of all categories of the clustering target is shown in Eq. 27.


(27) $$J=\sum_{k=1}^{K}\sum_{i=1}^{n}{||x_{i}-c_{k}||}^{2}$$

where *x*_*i*_ is the sample point and *c*_*k*_ is the clustering center.

**FIGURE 6:**
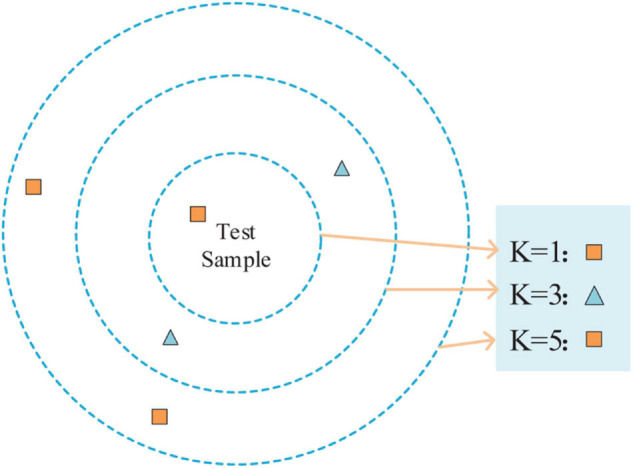
KNN classifier results with different values.

K nearest neighbor (Sreeshakthy et al., 2016) is a method, specifically supervised learning. For a given test sample, the closest *k* training samples in the training set are found using some distance measurements. After that, based on the information of these *k* ”neighbors,” the prediction is made. As shown in Figure 6, the different values of *k* result in different classification results.

The steps of the K-NN algorithm are as follows: (1) calculate the distance between the test samples and each training sample; (2) sort the test samples by distance incrementally; (3) *k* points with the smallest distance are selected as *k* ”neighbors”; (4) determine the occurrence frequency of the category of the first *k* points; and (5) the category with the most occurrence times in the first *k* points is returned as the prediction classification of the test sample.

Random forest (RF) (Breiman, 2001) is composed of several unrelated decision trees and the ultima classification result is up to the voting of the decision tree. The random forest has good noise resistance and is not easy to overfit. In the traditional decision tree, it is assumed that there are *d* attributes. When dividing the attribute, the optimal attribute is selected in the current node attributes. However, in a random forest, for each node of the decision tree, a subset containing *k* attributes is randomly selected from the attribute set of the node, and then the optimal attribute is selected from this subset for partition.

Softmax classifier (Zhang D. et al., 2020) can be used for both dichotomy and multi-classification. The SVM loss function is used to get a score and we can classify the original data by comparing the scores. Softmax can expand the score gap, even if there is little difference between the score results obtained by the score function. Through the Softmax classifier, the score gap can be further widened and the classification effect will be more obvious. Softmax classifier outputs the distribution probability of output categories, as shown in Eq. 28.


(28) $$soft\max\left(x_{i}\right)=\frac{\exp\left(x_{i}\right)}{\sum_{j=1}^{K}\exp\left(x_{j}\right)}$$

where *x*_*i*_ is the input, *Softmax*(⋅) is the Softmax activation function.

The Sigmoid classifier (Alhagry et al., 2017) is generally used for dichotomy. The definition of the Sigmoid activation function is shown in Eq. 29.


(29) $$Sigmoid\left(x_{i}\right)=\frac{1}{1+\exp\left(-x_{i}\right)}$$

where *Sigmoid*(⋅) represents the Sigmoid activation function. The output of the function is between 0 and 1. If a certain output is greater than a threshold, it is considered to belong to a certain category, otherwise, it is not.

## Review of Emotion Recognition

This section describes recent studies on EEG emotion recognition based on DEAP, SEED, and DREAMER public datasets. In total, 35 studies and experimental methods are described and the majority of the literature is based on deep learning for feature extraction and recognition of emotions. Finally, the effect of different methods on sentiment classification is summarized.

### Emotion Recognition Based on Database for Emotion Analysis Using Physiological Signals

Pane et al. (2019) proposed a strategy combining emotion lateralization and ensemble learning. Under four different channel sequences and combinations, time domain features, frequency domain features, and wavelet features of EEG signals were extracted. Then, the DEAP datasets were classified by random forest with a classification accuracy of 75.6%. Cheng et al. (2020) preprocessed EEG data and constructed a 2D frame sequence by using the spatial position relationship between channels and classified EEG emotion by deep forest. In the DEAP dataset, the average accuracy of valence and arousal was gained. Respectively, one was 97.69% and the other was 97.53%.

Ya et al. (2021) first extracted differential entropy to construct feature blocks and then used each segmented feature block as the input of a new deep learning model. The new deep learning model was constructed by fusing graph convolutional neural network (GCNN) and LSTM. Finally, extensive experiments are conducted for the DEAP dataset. Good results were obtained in experiments related to the subjects. Huang et al. (2021) proposed the Bi-Hemisphere Discrepancy Convolutional Neural Network Model (BiDCNN). It can effectively learn the different reaction patterns between the left and right hemispheres of the brain and construct a three-input and single-output network structure with three convolutional neural network layers. Putting the model into use in the DEAP datasets, its accuracy of potency and arousal is 94.38 and 94.72%, respectively.

Mokatren et al. (2021) used wavelet packet decomposition (WPD) to divide EEG signal into five sub-bands and extracted wavelet energy and wavelet entropy. The channel mapping matrix is constituted in accordance with the position of the EEG electrode. The extracted features are classified by CNN. In the DEAP dataset, the classification accuracy of valence and arousal was 91.85 and 91.06%, respectively. Moon et al. (2020) proposed a new classification system by using CNN for brain connectivity. In this method, Pearson correlation coefficient (PCC), phase locking value (PLV), and transfer entropy (TE) were used to complete the connectivity matrix and the effectiveness of the algorithm was verified on the DEAP dataset.

Liu J. et al. (2020) combined CNN, sparse autoencoder (SAE), and deep neural network (DNN) to propose a deep neural network for emotion recognition of EEG signals. On the DEAP dataset, the recognition accuracy of valence and arousal were 89.49 and 92.86%, respectively. Jca et al. (2020) converted the 1D chain-like EEG vector sequence into a 2D grid-like matrix sequence to extract the spatial correlation of the EEG signals between adjacent electrodes. Then, emotion can be recognized by combining the cascaded and parallel hybrid convolutional recurrent neural network. The binary classification experiments of valence and arousal emotion were carried out on the DEAP dataset and obtained accuracy of 93.64 and 93.26%, respectively. Yin et al. (2020) proposed a new locally-robust feature selection (LRFS) method. The method first used probability density to model the extracted EEG features. Then, the similarity of all density functions that existed in every two subjects was evaluated to describe the inter-individual consistency of EEG features, and the local robust EEG features were derived. Finally, ensemble learning was used to fuse selected features from a subset of multiple subjects. The accuracy of valence and arousal on the DEAP dataset was 67.97 and 65.10%, respectively.

Based on EEG segmentation for short-term change detection of facial markers, Tan et al. (2021) constructed a subject related short-term EEG emotion recognition framework based on spiking neural network (SNN) and optimized super parameters of the data representation of pulse coding and dynamic evolving SNN (deSNN). The accuracy of valence and arousal classification on the DEAP dataset was 67.76 and 78.97%, respectively. Salankar et al. (2021) put forward an emotion recognition algorithm dependent on empirical mode decomposition (EMD), which extracts the area, mean value, and central tendency measure of the elliptical region from second order difference plots (SODP) and classifies them by artificial neural networks (ANN). The accuracy rates of valence and arousal were 96.00 and 100.00%, respectively.

Zhou et al. (2020) proposed an EEG sample selection algorithm on the foundation of average Frechet distance to improve the sample quality. An EEG feature transfer algorithm based on transfer component analysis was developed to expand the sample size. Then, an EEG sample classification model based on echo state network (ESN) was constructed and the classification accuracy on the DEAP dataset was 68.06%. Pandey and Seeja (2019) first calculated the intrinsic mode functions (IMF) of each EEG signal by using the variational mode decomposition (VMD), then extracted the peak value of PSD and first difference according to the IMF, and finally input them to the deep neural network for classification. The accuracy of valence and arousal were 62.50 and 61.25%, respectively in the DEAP dataset. Liang et al. (2019) first extracted the features of EEG signal in the time domain, frequency domain, and wavelet domain, constructed hypergraph representation, and proposed an unsupervised learning classification method for classification on the DEAP dataset. The classification accuracy of valence and arousal were 54.45 and 62.34%, respectively.

Naser and Saha (2021) extracted the frequency domain features of EEG signals and proposed a feature-level fusion network for emotion classification by using dual-tree complex wavelet packet transform (DT-CWPT) and SVM. The accuracy of valence and arousal on the DEAP dataset was 69.33 and 69.49%, respectively. Şengür and Siuly (2020) first filtered and denoised EEG to extract rhythm. Then, the extracted rhythm signal was used to make a conversion from the rhythm signal to EEG rhythm images by continuous wavelet transform (CWT). Furthermore, the deep features of EEG rhythm images were extracted by convolution neural network, and the depth features were selected by MobileNetv2. Finally, LSTM was applied to emotion recognition. The accuracy of valence and arousal on the DEAP dataset were 96.1 and 99.6%, respectively.

Zhang S. et al. (2020) showed an emotion recognition method based on sample entropy (SE) and functional connection network. Firstly, WPT was used to decompose the EEG data of DEAP. Then, EEG features were constructed based on the functional connectivity network of phase synchronization index (PSI). Finally, RF was used for classification. The accuracy of valence and arousal was 86.67 and 88.58%, respectively on the DEAP dataset. Gao Y. et al. (2020) used information interaction between brain channels as a feature for classifying emotion states. In this paper, the transfer entropy (TE) relation matrix was combined with the Granger Causality (GC) and histogram of oriented gradient (HOG) to extract EEG features by image processing. The average classification accuracy of this method on the DEAP dataset was 88.93 and 95.21%, respectively.

Table 1 presents the conclusion of the above algorithms. This table summarizes the research methods and results of EEG emotion classification on the DEAP dataset. The feature extraction of EEG was carried out on the time domain, frequency domain, time-frequency domain, and deep learning domain. Researchers mainly used deep learning to identify and extract signals through various networks. Most of them are valence and arousal two-dimensional emotion models in emotion classification.

**TABLE 1:** Research results based on DEAP.

References	Method	Emotion	Accuracy	
			Valence	Arousal
Pane et al., 2019	Hybrid features extraction (time, frequency, and wavelet), RF	Valence	0.7560	—
Cheng et al., 2020	2D frame sequences, spatial position relationship, deep forest	Valence, Arousal	0.9769	0.9753
Ya et al., 2021	Differential entropy, graph convolutional neural network (GCNN), LSTM, Softmax	Valence, Arousal	0.8481	0.8527
Huang et al., 2021	Three different EEG feature matrices, Bi-hemisphere discrepancy convolutional neural network model (BiDCNN), Softmax	Valence, Arousal	0.9438	0.9472
Mokatren et al., 2021	Wavelet packet decomposition (WPD), wavelet energy, wavelet entropy, mapping matrix of the EEG channels, CNN	Valence, Arousal	0.9185	0.9106
Moon et al., 2020	Phase-locking value (PLV), Pearson correlation coefficient (PCC), transfer entropy (TE), connectivity matrices, CNN, Softmax	Valence	0.8736	—
Liu J. et al., 2020	Convolutional Neural Network (CNN), Sparse Autoencoder (SAE), Deep Neural Network (DNN), Sigmoid	Valence, Arousal	0.8949	0.9286
Jca et al., 2020	1D chain-like EEG vector, 2D meshlike matrix sequences, cascaded and parallel hybrid convolutional recurrent neural network, Softmax	Valence, Arousal	0.9364	0.9326
Yin et al., 2020	Locally-robust feature selection (LRFS), emotion classifier committee, ensemble learning	Valence, Arousal	0.6797	0.6510
Tan et al., 2021	Dynamic evolving SNN (deSNN), spike-timing-dependent plasticity (STDP)	Valence, Arousal	0.6776	0.7897
Salankar et al., 2021	Empirical Mode Decomposition (EMD), second order difference plots (SODP), artificial neural networks (ANN)	Valence, Arousal	0.9600	1.0000
Zhou et al., 2020	Frechet distance, echo state network (ESN), transfer learning (TL)	Valence	0.6806	—
Pandey and Seeja, 2019	Peak value of PSD, first difference, variational mode decomposition (VMD), intrinsic mode functions (IMF), deep neural network	Valence, Arousal	0.6250	0.6125
Liang et al., 2019	Statistical features (7 features), Hjorth features (3 features), fractal dimension (FD), hypergraph theory, unsupervised learning	Valence, Arousal	0.5445	0.6234
Naser and Saha, 2021	Dual-tree complex wavelet packet transform (DT-CWPT), feature-level fusion, SVM	Valence, Arousal	0.6933	0.6949
Şengür and Siuly, 2020	Continuous wavelet transform (CWT), MobilNetv2, LSTM	Valence, Arousal	0.9610	0.9960
Zhang S. et al., 2020	Sample entropy (SE), Functional connection network, Wavelet Packet Transform (WPT), Phase synchronization index (PSI), global clustering coefficient, local clustering coefficient, etc., phase synchronization index, FR	Valence, Arousal	0.8667	0.8858
Gao Y. et al., 2020	Histogram of Oriented Gradient (HOG), Granger Causality (GC), Transfer Entropy (TE), SVM	Arousal, Valence	GC:0.8893	
		Liking, Dominance	TE:0.9521	

*”—” means that this experiment was not done in this research.*

### Emotion Recognition Based on SEED

Asa et al. (2021) extracted six features of each wavelet sub-band based on tunable Q wavelet transform (TQWT) and used six different methods for dimensionality reduction. Finally, rotation forest ensemble (RFE) was used for different classification algorithms to classify the SEED dataset and the SVM classifier achieved the highest classification accuracy of 93.1%. Wei et al. (2020) decomposed the original EEG into five sub-bands through DT-CWT. Then, these sub-band features of time domain, frequency domain, and non-linear were extracted. At last, three integration tactics were adopted to integrate the simple recurrent units (SRU) model to obtain final classification performance.

Topic and Russo (2021) proposed topographic feature maps (TOPO-FM) and holographic feature maps (HOLO-FM) based on nine EEG signal features. Then, the features were extracted and fused with CNN. Wang et al. (2019) determined the pattern of functional connectivity related to emotion in the application of PLV connectivity of EEG signals. In different emotional states, the internal relationship between EEG channels was expressed. The neural network was used for training to distinguish emotional states, and the classification accuracy on the SEED dataset was 84.35%. Li et al. (2021) proposed a kind of transferable attention neural network (TANN) for EEG emotion recognition. The network took into account the internal structure information of electrodes and adaptively highlights the data and samples of transferable EEG brain regions through local and global attention mechanisms to learn emotion recognition information. The accuracy in the SEED data set was 84.41%.

Wang F. et al. (2020) proposed a concept of electrode-frequency distribution maps (EFDM) based on short-time Fourier transform. Then, four residual-block-based CNN was constructed with EFDM as input for emotion classification. The classification accuracy on the SEED dataset was 90.59%. Asghar et al. (2021) firstly decomposed the original EEG signal to empirical coefficients by using intensive multivariant empirical mode decomposition (IMEMD). Then, the empirical coefficients were analyzed by collecting all the information in the time domain and frequency domain by using complex continuous Wavelet transform (CCWT). Finally, combining with three deep neural networks, SVM was used for classification. The classification accuracy rate on the SEED dataset reached 96.3%.

Lu et al. (2019) proposed a new pattern learning framework based on dynamic entropy. Firstly, continuous entropy was extracted from EEG. Then, the continuous entropy is concatenated to form a feature vector and pattern learning based on dynamic entropy can realize the emotion recognition across individuals to get excellent emotion recognition precision. The accuracy of the two kinds of emotion recognition was 85.11% when tested on the SEED dataset. Rahman et al. (2020) first used PCA that can reduce the dimension of EEG signal, then extracted the standard deviation, mean absolute deviation, and power spectral density of EEG signal as classification features, and used t-statistic to select distinctive features. Finally, SVM and other classifiers were used to recognize emotions on the SEED dataset and the accuracy reached 84.3%. Joshi (2021) proposed an emotion recognition algorithm based on the linear formulation of differential entropy (LFDE) and bi-directional long short-term memory (BiLSTM) network. The average classification accuracy of this algorithm on the SEED dataset was 80.64%.

An emotion recognition algorithm based on conditional generative adversarial network (cGAN) was proposed by Fu et al. (2021), which realized fine-grained estimation and visualization of emotion based on EEG. Two experiments were carried out on the SEED dataset and the average classification accuracy was 92.02 and 82.14%. Cheah et al. (2021) proposed an EEG emotion recognition algorithm based on residual networks (ResNet), which achieved 93.42% accuracy on the SEED dataset.

The above algorithms are summarized in Table 2. This table summarizes the algorithms and results of EEG emotion classification by using the SEED dataset. The feature extraction of EEG is completed in four aspects: time domain, frequency domain, time-frequency domain, and deep learning. In terms of signal classification, it is mainly based on positive, negative, and neutral emotions.

**TABLE 2:** Research results based on SEED.

References	Method	Emotion	Accuracy
Asa et al., 2021	Mean absolute value, Average power, etc., A tunable Q wavelet transform (TQWT), rotation forest ensemble (RFE), SVM, K-NN	Negative, Positive, Neutral	0.9310
Wei et al., 2020	Mean absolute value (MAV), PSD, etc. Simple Recurrent Units (SRU), Dual-tree Complex Wavelet Transform (DT-CWT)	Negative, Positive, Neutral	0.8313
Topic and Russo, 2021	Hjorth activity, mobility and complexity, peak-to-peak, etc. 9 features, topographic feature map (TOPO-FM), holographic feature map (HOLO-FM), CNN, SVM	Negative, Positive, Neutral	0.7311
Wang et al., 2019	Phase-locking value graph convolution neural networks (P-GCNN)	Negative, Positive, Neutral	0.8435
Li et al., 2021	Intrinsic structural information of electrodes, RNN, transferable attention neural network (TANN), local attention, global attention	Negative, Positive, Neutral	0.8441
Wang F. et al., 2020	Short-time Fourier transform (STFT), Electrode-frequency distribution maps (EFDM), residual block based deep CNN	Negative, Positive, Neutral	0.9059
Asghar et al., 2021	Intensive multivariate empirical mode decomposition (IMEMD), Complex Continuous Wavelet Transform (CCWT), deep neural network, SVM	Negative, Positive, Neutral	0.9630
Lu et al., 2019	Dynamic entropy, SVM	Negative, Positive	0.8511
Rahman et al., 2020	Standard deviation, mean absolute deviation, power spectral density, PCA, t-statistics, SVM	Positive, Negative, Neutral	0.8430
Joshi, 2021	Linear Formulation of Differential Entropy (LFDE), Bi-directional Long Short-Term Memory (BiLSTM)	Positive, Negative, Neutral	0.8064
Fu et al., 2021	Conditional generative adversarial network(cGAN)	Positive, Negative	0.9202
Cheah et al., 2021	Residual networks (ResNet), Visual Geometry Group(VGG)	Positive, Negative, Neutral	0.9342

*”—” means that this experiment was not done in this research.*

### Emotion Recognition Based on DREAMER

Because of asymmetry between the left and right hemispheres of the brain, discriminative features are obtained. With it as the foundation, Cui et al. (2020) proposed a feature extraction algorithm. Emotion was then identified by constructing an end-to-end asymmetric regional-asymmetric convolution neural network (RACNN). The valence and arousal accuracy of the algorithm on the DREAMER dataset were 95.55 and 97.01%, respectively. Cheng et al. (2020) proposed an EEG emotion recognition algorithm based on the multi-grained cascade forest (gcForest) model. The average classification accuracy of this algorithm on the DREAMER dataset reached 89.03, 90.41, and 89.89%, respectively.

Liu Y. et al. (2020) proposed a multi-level features guided capsule network (MLF-CapsNet) for emotion recognition based on multi-channel EEG. Its function was to simultaneously extract features from raw EEG signals and determine emotional states. The average accuracy rates of valence, arousal, and dominance in the DREAMER dataset were 94.59, 95.26, and 95.13%, respectively. Wang Y. et al. (2020) proposed a new EEG emotion recognition algorithm based on domain adaptive SPD matrix network (daSPDnet). Making use of feature adaptation with distribution confusion and the sample adaptation with centroid alignment, this algorithm computed the sample point diffusion matrix based on covariance and combined prototype learning with the Riemannian metric. The shared emotional representation among different subjects can be captured successfully. The average accuracy rates of the proposed method were 67.99, 76.57, and 81.77% on the DREAMER dataset, respectively.

Dm et al. (2021) proposed a multi-channel EEG emotion recognition algorithm that takes advantage of a rhythm-specific convolutional neural network. The accuracy of valence, arousal, and dominance in the DREAMER dataset were 97.17, 96.81, and 97.24%, respectively. Song et al. (2018) gave a multi-channel emotion recognition method based on a new dynamic graph convolutional neural network (DGCNN). The average recognition accuracy of valence, arousal, and dominance classification in the DREAMER database were 86.23, 84.54, and 85.02%, respectively.

The conclusion of the above algorithms is shown in Table 3. This table summarizes the algorithms and results of EEG emotion classification on the DREAMER dataset. At present, there are relatively few experiments using this dataset, most of which are comparative experiments. The researchers mainly use deep learning to extract features of EEG. In the aspect of signal classification, it includes a two-dimension emotion classification model of valence and arousal and a three-dimension emotion classification model of valence, arousal, and domination.

**TABLE 3:** Research results based on DREAMER.

References	Method	Emotion	Accuracy		
			Valence	Arousal	Dominance
Cui et al., 2020	Regional-Asymmetric Convolution Neural Network (RACNN), Asymmetric Differential Layer (ADL), softmax	Valence, Arousal	0.9555	0.9701	—
Cheng et al., 2020	2D frame sequences, spatial position relationship, deep forest	Valence, Arousal	0.8903	0.9041	0.8989
Liu Y. et al., 2020	Multi-level features guided capsule network (MLF-CapsNet)	Valence, Arousal, Dominance	0.9459	0.9526	0.9513
Wang Y. et al., 2020	SPD matrix network (daSPDnet)	Valence, Arousal, Dominance	0.6799	0.7657	0.8177
Dm et al., 2021	Multi-channel, rhythm selection, CNN, softmax	Valence, Arousal, Dominance	0.9717	0.9681	0.9724
Song et al., 2018	Dynamic graph convolution neural network (DGCNN), Softmax	Valence, Arousal, Dominance	0.8623	0.8454	0.8502

*”—” means that this experiment was not done in this research.*

## Conclusion

This paper summarizes existing research about emotion recognition based of EEG. Firstly, the mechanism of EEG, emotion trigger mode, and classification model are introduced in detail. Then, we elaborate the existing EEG emotion recognition algorithms from three aspects, which are feature extraction, feature selection, and classifier. Finally, the results of various emotion classification methods are discussed and compared by reviewing the literature. In the practical application of EEG emotion recognition, many problems are still waiting to be solved, such as few emotion categories and datasets, which are also the focus of future research in this field.

## Author Contributions

YZ performed the computer simulations. HL analyzed the data. YZ and HL wrote the original draft. YL and XK revised and edited the manuscript. All authors confirmed the submitted version.

## Conflict of Interest

The authors declare that the research was conducted in the absence of any commercial or financial relationships that could be construed as a potential conflict of interest.

## Publisher’s Note

All claims expressed in this article are solely those of the authors and do not necessarily represent those of their affiliated organizations, or those of the publisher, the editors and the reviewers. Any product that may be evaluated in this article, or claim that may be made by its manufacturer, is not guaranteed or endorsed by the publisher.
